# Genetic variation in taste receptor pseudogenes provides evidence for a dynamic role in human evolution

**DOI:** 10.1186/s12862-014-0198-8

**Published:** 2014-09-13

**Authors:** Davide Risso, Sergio Tofanelli, Gabriella Morini, Donata Luiselli, Dennis Drayna

**Affiliations:** National Institute on Deafness and Other Communication Disorders, NIH, Bethesda, MD 20892 USA; Department of BiGeA, Laboratory of Molecular Anthropology and Centre for Genome Biology, University of Bologna, via Selmi 3, 40126 Bologna, Italy; Department of Biology, University of Pisa, Via Ghini 13, 56126 Pisa, Italy; University of Gastronomic Sciences, Piazza Vittorio Emanuele 9, Bra, Pollenzo, 12042 CN Italy

**Keywords:** Bitter taste, Evolution, Genetic polymorphisms, Pseudogenization, *TAS2Rs*

## Abstract

**Background:**

Human bitter taste receptors are encoded by a gene family consisting of 25 functional *TAS2R* loci. In addition, humans carry 11 *TAS2R* pseudogenes, some of which display evidence for substantial diversification among species, showing lineage-specific loss of function. Since bitter taste is thought to help prevent the intake of toxic substances, diversity at *TAS2R* genes could reflect the action of natural selection on the ability to recognize some bitter compounds rather than others. Whether species-specific variation *in TAS2R* pseudogenes is solely the result of genetic drift or whether it may have been influenced by selection due to different feeding behaviors has been an open question.

**Results:**

In this study, we analyzed patterns of variation at human *TAS2R* pseudogenes in both African and non-African populations, and compared them to those observable in nonhuman primates and archaic human species. Our results showed a similar worldwide distribution of allelic variation for most of the pseudogenes, with the exception of the *TAS2R6P* and *TAS2R18P* loci, both of which presented an unexpected higher frequency of derived alleles outside Africa. At the *TAS2R6P* locus, two SNPs were found in strong linkage disequilibrium (r^2^ > 0.9) with variants in the functional *TAS2R5* gene, which showed signatures of selection. The human *TAS2R18P* carried a species-specific stop-codon upstream of four polymorphic insertions in the reading frame. SNPs at this locus showed significant positive values in a number of neutrality statistics, and age estimates indicated that they arose after the homo-chimp divergence.

**Conclusions:**

The similar distribution of variation of many human bitter receptor pseudogenes among human populations suggests that they arose from the ancestral forms by a unidirectional loss of function. However we explain the higher frequency of *TAS2R6P* derived alleles outside Africa as the effect of the balancing selection acting on the closely linked *TAS2R5* gene. In contrast, *TAS2R18P* displayed a more complex history, suggesting an acquired function followed by a recent pseudogenization that predated the divergence of human modern and archaic species, which we hypothesize was associated with adaptions to dietary changes.

**Electronic supplementary material:**

The online version of this article (doi:10.1186/s12862-014-0198-8) contains supplementary material, which is available to authorized users.

## Background

The perception of bitter taste is thought to have evolved as a protection from toxic and harmful foods [1,2]. However, not all bitter compounds evoke the same aversive reaction in different species and, in particular, among primates [3]. Therefore, it has been hypothesized that consumption or rejection response depends upon the relative occurrence of bitter and potentially toxic compounds in an animal’s diet [4]. The number of different bitter taste receptors varies greatly across species [5], but all of them belong to a family of seven-transmembrane G protein coupled receptors (GPCRs) known as *TAS2Rs* or *T2Rs*. In humans, this gene family is encoded by 25 functional *TAS2R* loci, which reside on chromosomes 5, 7 and 12 [6]. In addition to these genes, humans also carry 11 *TAS2R* pseudogenes [7]. Polymorphisms in these genes have been shown to modulate the taste response to different compounds, both natural and synthetic [8-10], and showed evidence of evolutionary pressures [11-13], highlighting the importance of studying both *TAS2R* genes and pseudogenes.

Pseudogenes are considered genomic fossils, classically defined as genomic loci with sequence similarity to functional genes, but lacking coding potential [14,15], often due to disruptive mutations such as frameshifts, premature stop codons and deletions. However, recent studies have demonstrated that some pseudogenes may likely have a function, providing evidence for their plasticity and a dynamic role in evolution [16-18]. A number of authors have described these events with a “less-is-more” hypothesis, suggesting that gene loss, or pseudogenization, may serve as an engine of evolutionary change, especially in human evolution [19,20]. In fact, previous phylogenetic analyses demonstrated that remarkable variation exists in both *TAS2Rs* genes and pseudogenes in different lineages, suggesting that the ability to perceive bitterness may be mostly due to the repertoire of *TAS2R* genes [21,22]. For instance, the mouse genome contains 33 functional *TAS2Rs* loci but only three pseudogenes, and the evolutionary relationships between human and mouse genes were shown to fall into three categories, depending on their orthology: 1) one-to-one orthology; 2) one-to-multiple orthology; 3) multiple-to-one orthology [23,15,7]. In contrast, zebrafish and chicken have only four and three *TAS2R* loci, respectively [24,5]. Comparative analyses showed that primate *TAS2Rs* had a higher ratio of nonsynonymous/synonymous substitutions and a lower selective pressure on this gene family compared to rodents [25,26].

To better understand the evolutionary mechanisms underlying taste receptor pseudogenization events in the human lineage, we performed a survey of the human genetic variation at nine human TAS2RP loci. We then compared these to the homologous pseudogenes present in archaic human forms (i.e. Neandertal and Denisovan), as well as in other five primate species, Chimpanzee, Gorilla, Orangutan, Gibbon and Mouse Lemur. We examined the intra- and inter-species variation patterns to search for possible footprints of natural selection at these loci, with the goal of understanding the potential adaptive role of taste receptor pseudogenes in the evolutionary history of modern humans.

## Methods

### Genes and data sets

Nine human *TAS2R* pseudogenes were selected from the literature and are shown in Table 1. Modern human variants located in these genes, together with their surrounding genomic regions (+/− 100 kbp), were retrieved from the 1000 Genomes Project PHASE I database [27,28], which provided data on a total number of 1,092 individuals belonging to 14 different populations. Neandertal and Denisovan variants/sequences were retrieved at the UCSC Table Browser [29,30]. Pseudogene sequences in humans (*Homo sapiens*), five other primates (*Pan troglodytes*, *Gorilla gorilla*, *Pongo abelii*, *Nomascus leucogenys* and *Microcebus murinus*) and one rodent (*Mus musculus*), were obtained from the Ensembl Genome Browser [31,32]. This project was approved by the NIH protocol 01-DC-0230, reviewed by the NIH/NINDS CNS Blue Panel IRB and complied with the Helsinki Declaration of Ethical Principles.

**Table 1:** ***TAS2R*** **pseudogenes chromosomal positions (GRCh37)**

**Pseudogene**	**Chromosome**	**Position**
*TAS2R2P*	7	12,530,72-12,531,630
*TAS2R6P*	7	141,487,614-141,488,440
*TAS2R62P*	7	143,134,127-143,135,066
*TAS2R12P*	12	11,047,542-11,048,481
*TAS2R15P*	12	11,117,024-11,117,951
*TAS2R18P*	12	11,311,384-11,312,293
*TAS2R63P*	12	11,200,931-11,201,855
*TAS2R64P*	12	11,229,368-11,231,770
*TAS2R67P*	12	11,332,272-11,333,061

### Haplotype analyses

Haploview [33] was used to identify Linkage Disequilibrium (LD) patterns and haplotype blocks at the selected loci using the solid spine approach. Genealogical relationships among inferred haplotypes were constructed using the median-joining algorithm implemented in the Network 4.5 program [34]. Haplotypes distribution across human populations were investigated using PLINK v.1.07 [35].

### Population genetics analyses

Arlequin v.3.5 [36] was used to compute summary statistics, such as nucleotide diversity (π), estimated heterozygosity (EH) and number of polymorphic sites (PS). Chi square-tests were performed to compare allele frequencies among different populations and the adopted significance threshold was adjusted using the Bonferroni correction (i.e. adjusted p = p value X number of individual tests). Metric Multidimensional Scaling (MDS) analyses based on the obtained F_ST_ values, were performed with STATISTICA v. 6.0 (Stat-Soft Inc, Tulsa, OK).

### Phylogenetic studies

MUSCLE v.3.3 [37] (Edgar 2004) was used to perform multi-alignments of the examined sequences. A maximum likelihood tree was constructed with MEGA v.6.0 [38] using the Tamura-Nei substitution model. To assess the relative support for each clade, bootstrap values were calculated from 10.000 analysis replicates, and the cut-off point for bootstrap replication was 50%.

### Neutrality test and age estimates

To test whether patterns of allele/haplotype frequencies and tree topology were consistent with neutral expectations, we performed three neutrality tests. For this purpose, the DNASP package [39] was used to calculate Tajima’s D and Fu’s FS values at each locus. Since deviations found using these tests could be caused by selection and/or demographic processes (e.g. population expansion and/or bottlenecks), we also performed the Li’s MFDM test [40], which is more robust in the presence of population size changes. Finally, GENETREE [41,42] was used to infer the estimated age of selected variants and the time of the most recent common ancestor (TMRCA).

## Results

### Sequence variation

A total of 47 single nucleotide polymorphisms (SNPs) (Additional file 1: Table S1), annotated according to dbSNP Build 137, were observed in the nine selected pseudogenes. 32 of them had a minor allele frequency (MAF) above 5%. Unexpected patterns of genetic variation were observed for two pseudogenes, *TAS2R18P* and *TAS2R6P*, in populations from different continents. In particular, the derived alleles at four *TAS2R18P* SNPs (*rs2290318*, *rs2290319*, *rs61928604* and *rs61928603*) showed significantly increased frequency (0.5) in non-African populations compared to African populations (0.053) (Fisher’s exact test, adjusted p < 0.01). Similarly, the derived alleles of *TAS2R6P* polymorphisms *rs1859645* and *rs11761380* showed a significantly different distribution (0.24 vs. 0.57) between Africa and other continents (Fisher’s exact test, adjusted p < 0.01). Both Neandertal and Denisovan genomes carried the derived alleles of the *TAS2R18P* SNPs *rs2290318*, *rs2290319*, *rs61928604* and *rs61928603*. For the *TAS2R6P* SNPs *rs1859645* and *rs11761380*, the Denisovan genome showed ancestral alleles, while the Neandertal genome was heterozygous at both SNPs. In addition, derived alleles of two SNPs located in *TAS2R67P* (*rs319269* and *rs34648613*) and one in *TAS2R64P* (*rs68071847*) were more frequent in African populations with respect to non-African ones (0.68 vs. 0.11, Fisher’s exact test, adjusted p < 0.01), and were also present in both Neandertal and Denisovan genomes. Finally, the derived allele of one SNP located in *TAS2R63P* (*rs2597986*) was present only in a few African individuals (1.58%), as well as in the two archaic species.

### Haplotype structure

A total of eight haplotype blocks (Additional file 2: Figure S1 A-B) were inferred in the nine studied pseudogenes. Five of them (H1-H5) were on chromosome 7, with the remaining three (H6-H8) located on chromosome 12. H5, which contained *TAS2R6 rs11761380* and *rs1859645*, and H6, made up of *TASR18 rs2290318* and *rs2290319*, showed high level of LD (R^2^ = 1) and differed significantly across human populations (Table 2), with African populations carrying a lower percentage of derived alleles at these loci. The other haplotype blocks (H1, H2, H3, H4, H7 and H8) showed no significant differences among continents.

**Table 2:** **Comparisons of** ***TAS2R*** **pseudogene haplotype blocks among the studied populations**

**HBlock**	**Chr**	**PseudoGene**	**SNP1**	**SNP2**	**MA1**	**MA2**	**F_AFR**	**F_ASN**	**F_EUR**	**F_AMR**	**Comparison**	adjusted P-value*
H5	7	TAS2R6P	rs1161380	rs1859645	C	G	0,24	0,71	0,46	0,54	AFR/EUR	1,16E-15
											AFR/AMR	1,5E-18
											AFR/ASN	1,5E-17
											EUR/ASN	2,11E-09
H6	12	TAS2R18P	rs2290318	rs2290319	C	A	0,05	0,74	0,35	0,41	AFR/EUR	2,13E-13
											AFR/AMR	1,03E-09
											AFR/ASN	1,11E-20
											EUR/ASN	1,07E-08
											ASN/AMR	2,21E-06

Hblock, Haplotype block; Chr, Chromosome; MA, Minor Allele; F, Frequence.

*after Bonferroni correction

AMR, Latin Americans; AFR, Africans, EUR, Europeans; ASN, Asians.

To test the significance of the observed structure, the distribution of the H5 and H6 haplotypes in human populations was also investigated by means of Analysis of Molecular Variance (AMOVA). For both *TAS2R6P* and *TAS2R18P*, most of variation was accounted for by differences within populations (86.67% and 72.07%, respectively), with a smaller percentage attributed to differences among (13.2% and 27.12%) and within groups (0.13% and 0.81%). The global F_ST_ values were 0.13 for *TAS2R6P* and 0.28 for *TAS2R18P*, while those related to H5 and H6 haplotypes were 0.14 and 0.31, respectively.

We constructed median-joining networks for the *TAS2R6* and *TAS2R16* pseudogenes in order to better understand the relationships between the inferred haplotypes. The resultant topologies (Additional file 3: Figure S2 A-B) identified of two major clusters defined by the presence of either the derived (GC for *TAS2R6P*, CA for *TAS2R18P*) or ancestral (AA for *TAS2R6P*, GC for *TAS2R18P*) alleles at the H5 and H6 haplotypes.

### Summary statistics and population structure

The pattern of diversity shown in most of these pseudogenes was in accordance with the usual distribution of human genetic variation [38], where the diversity is higher in African populations than in non-African ones. However, a different situation was observed in *TAS2R6P* and *TAS2R18P*. African groups (i.e. ASW, LWK and YRI) showed lower values of both nucleotide diversity (π) and estimated heterozigosity (EH) at the*TAS2R18P* locus. With the exception of the ASW group, the same pattern was observed for the *TAS2R6P* gene. The number of polymorphic sites (PS) was similar in all the studied populations (Additional file 4: Table S2 A-B).

**Figure 1:**
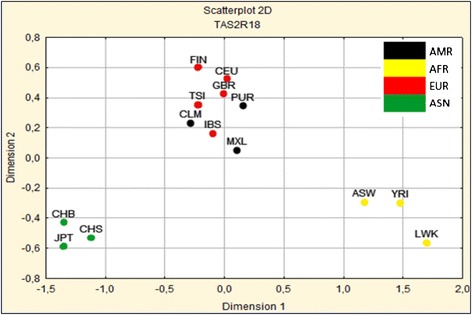
**Two-dimensional scaling of the F**_**ST**_ **distance matrix calculated for** ***TAS2R18P*** **.** AMR, Latin Americans; AFR, Africans, EUR, Europeans; ASN, Asians; YRI, Yoruba from Ibadan (Nigeria); LWK, Luhya from Webuye (Kenya); ASW, people with African ancestry from Southwest United States; IBS, Iberian populations from Spain; TSI, Tuscans from Italy; CEU, Utah residents with Northern and Western European ancestry; GBR, British from England and Scotland; FIN, Finnish; PUR, Puerto Ricans; CLM, Colombians from Medellin; MXL, people with Mexican ancestry from Los Angeles; JPT, Japanese from Tokyo; CHB, Han Chinese from Beijing; CHS, Han Chinese from Southern China.

To explore the population structure at these two outlier loci, we calculated pairwise Wright’s F_ST_ indices as a measure of genetic differentiation among different groups. A multidimensional scaling (MDS) metric was then used to plot the obtained F_ST_ values. This produced a clear distinction between continental populations for the observed variation. As shown in Figure 1, *TAS2R18P* was separated from African and Asian populations along the first dimension, with a distinct cluster, containing the European and Latin American (admixed) populations, occupying an intermediate position between them. Variation at the *TAS2R6P* locus showed a similar pattern of population structure among continental clusters (Additional file 5: Figure S3).

### Inter-specific comparison

**Figure 2:**
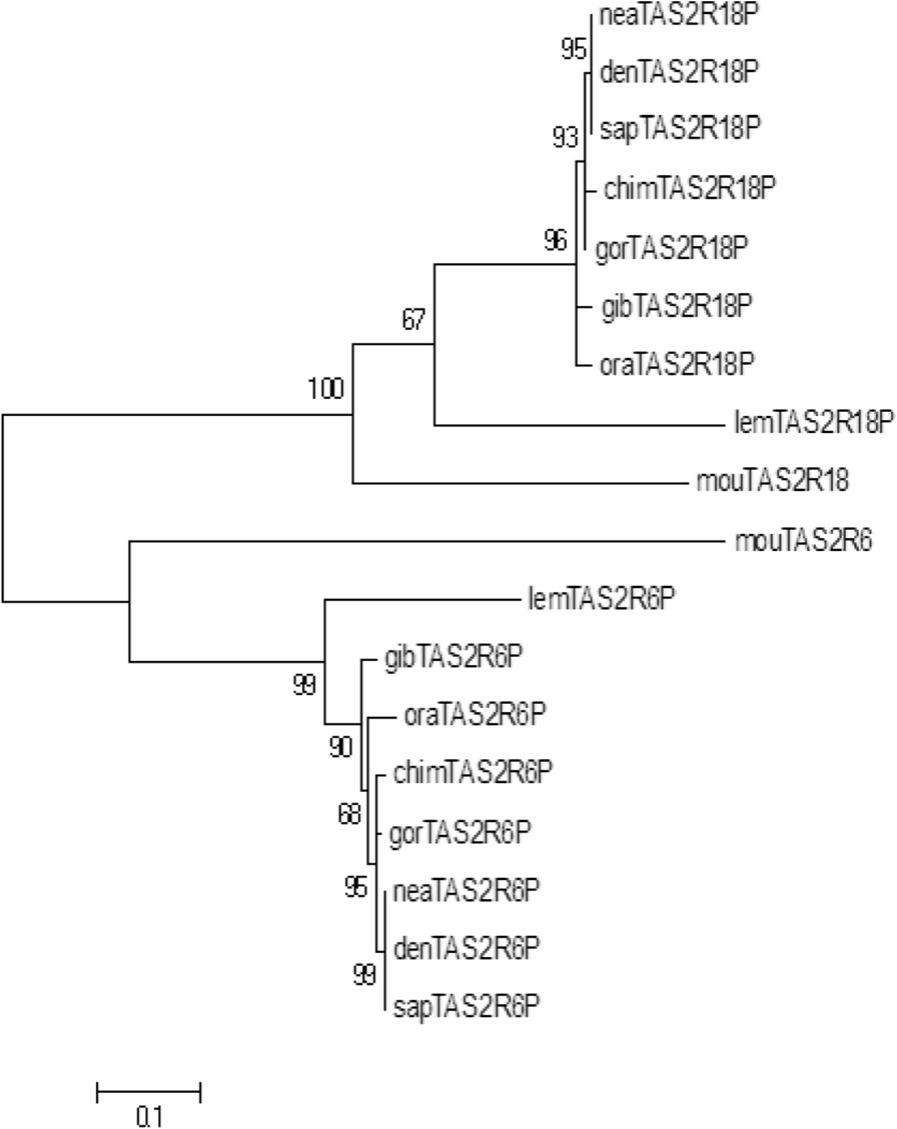
**Maximum Likelihood tree of** ***TAS2R18P*** **and** ***TAS2R6P*** **.** Bootstrap values are shown on the branch forks, the scale bar corresponds to the branch length and indicates 0.1 amino acid substitution per site. *mou*, Mouse; *lem*, Lemur; *gib*, Gibbon; *ora*, Orangutan; *gor*, Gorilla; *chi*, Chimpanzee; *nea*, Neadertal; *den*, Denisova; *sap*, Sapiens.

We also created a phylogenetic tree using the nucleotide sequences of *TAS2R6P* and *TAS2R18P* to understand the phylogenetic relationships between both modern and archaic humans and other primates. As shown in Figure 2, *TAS2R6P* sequences in Prosimians, Hylobatidae and Hominidae are closely related, sharing a common ancestor when compared to the outgroup sequence (i.e. *Mus musculus*) and showing short branch lengths of the tree, suggesting an ancient pseudogenization event at this locus. Longer branch lengths in Prosimians and Anthropoidea after their divergence may indicate that the pseudogenization at *TAS2R18P* locus occurred independently in the two lineages. It should be noted that these conclusions might be affected by problems with ortholog detection, considering the high dynamism and extreme complexity of this gene family.

However, DNA sequence similarities confirmed these results, showing a high percentage of *TAS2R6P* identity between Hominidae and Hylobatidae (>95%) and Prosimians, Hominidae and Hylobatidae (>75%). *TAS2R18P* sequences showed a lower degree of similarities (<70%) between primates and Prosimians (Additional file 6: Table S3 A-B).

**Figure 3:**
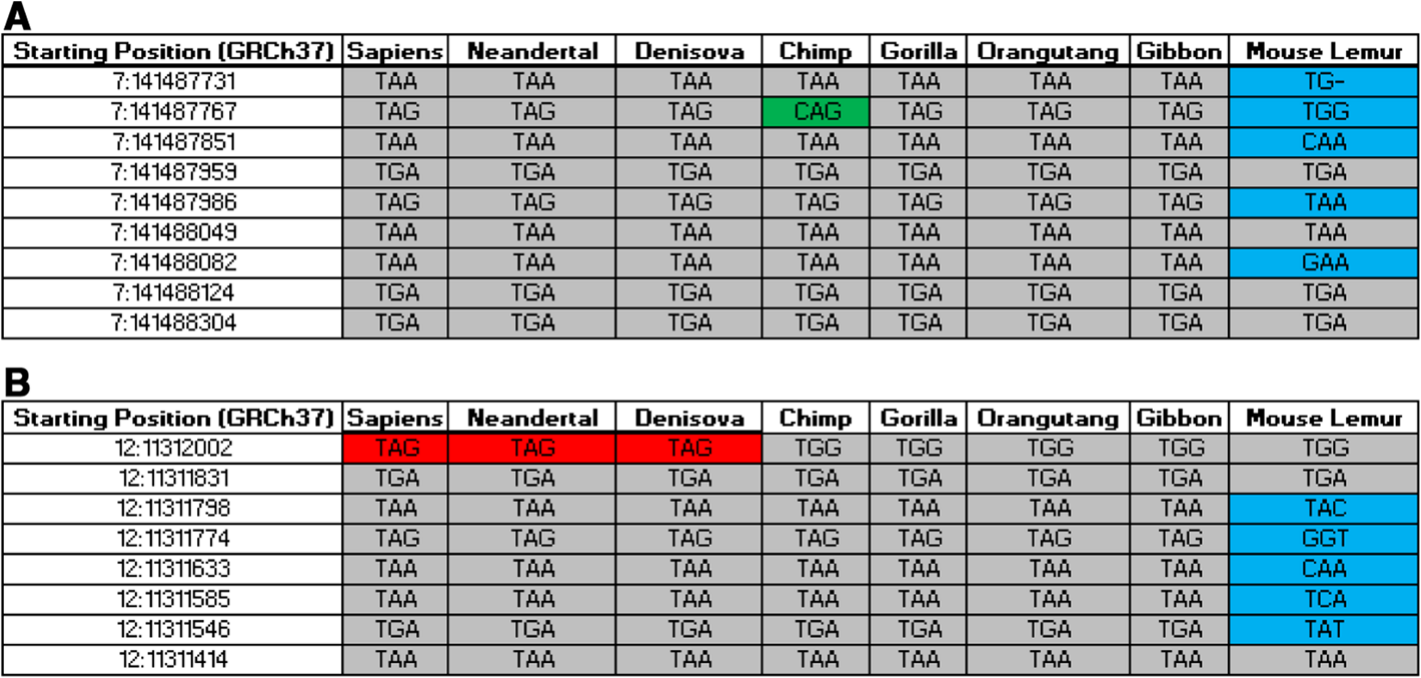
**Multi-alignment of** ***TAS2R6P*** **(A) and** ***TAS2R18P*** **(B) stop-codon sequences.** Conserved sequences are indicated in grey. Changes between humans and other primates are denoted in red, changes between Prosimians and primates are noted in blue, and the change between Chimp and other primates is noted in green.

In addition, multi-alignments of *TAS2R6P* and *TAS2R18P* produced results in agreement with the findings described above. As shown in Figure 3A, *TAS2R6P* shows a high degree of conservation among all the species examined. Some of its stop codons, such as those at positions 7:141487959 and 7:141488049, are shared with the earlier representatives of this lineage. Besides species-specific changes found in lemur, and in agreement with the ancient time of the Strepsirrhini-Haplorrhini divergence (64 Mya), only one unique stop-loss mutation (TAG > CAG) was found in the chimpanzee *TAS2R6P*. All the human species (i.e. H. sapiens, Neandertal and Denisova) shared a fixed stop-gain mutation (TGG > TAG) on *TAS2R18P* (Figure 3b), which was not present in other primates that carry other stop-codon mutations at this locus and show a high degree of conservation between Hominidae and Hylobatidae. The reduced conservation between Prosimians and other primates is consistent with the view that the two pseudogenization events appeared at different times during their evolutionary histories.

**Figure 4:**
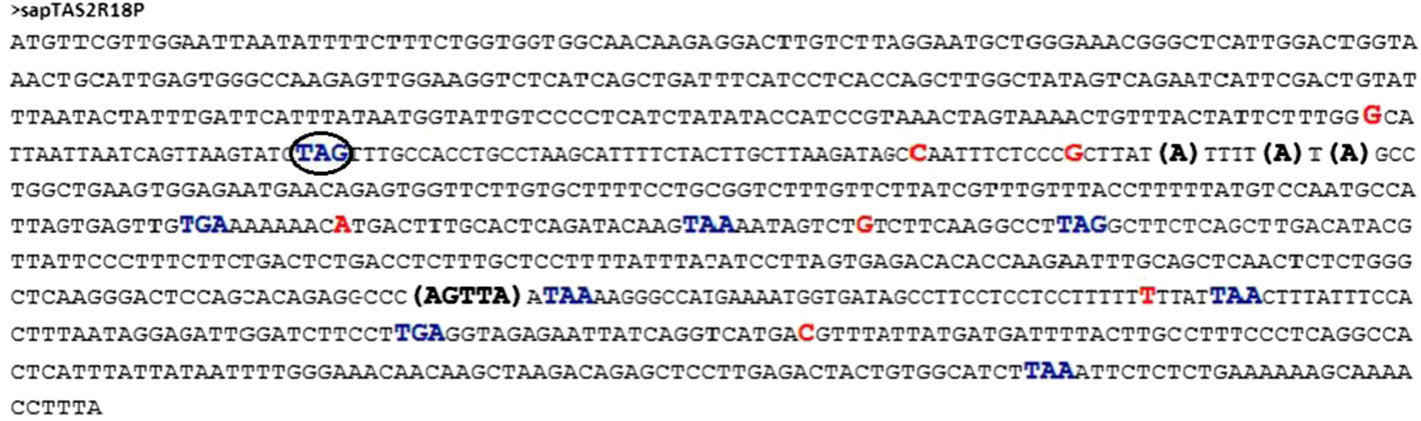
**Human** ***TAS2R18P*** **sequence.** Insertions are in bold, polymorphic SNPs in red and stop-codons in blue. Human-specific stop-codon is circled.

To better understand the species-specific changes that occurred in the *TAS2R18P* locus, the human exonic region of this gene was investigated (Figure 4). Four polymorphic insertions (*rs10619393*, *rs373807934*, *rs66547287* and *rs113657094*) were identified in the reading frame, upstream of the seven stop-codons shared across species and downstream of the human-specific stop codon. One of them (*rs113657094*) showed clear differential distribution among continental clusters of populations, with Africans displaying a significantly lower frequency of the A insertion (42%) compared to other ethnic groups (86% in Latin Americans, 88% in Europeans and 97% in Asians) (Fisher’s exact test, adjusted p < 0.01). For comparison, the *TAS2R6P* sequence was also examined and neither insertions nor other structural variants were found.

### Relationships with functional genes

A 200 kb (100 kb in both 5’ and 3’ directions) region surrounding both *TAS2R6P* and *TAS2R18P* was explored for linkage disequilibrium patterns. The *TAS2R6P rs11761380* and *rs1859645* polymorphisms were found to be in strong LD (r^2^ > 0.9) with SNPs *rs62477710*, *rs10952507* and *rs6962558* that reside in the functional *TAS2R5* gene (Additional file 7: Table S4). In contrast, *TAS2R18P* showed no r^2^ values above 0.3 between variants. In addition, we analyzed the sequences of all the genes found in this enlarged genomic interval and compared them to the pseudogene sequences, in order to document the sequence similarities between pseudogenes and functional genes. *TAS2R6P* shares a common ancestor with the functional *TAS2R5* gene (62.59% identity), while *TAS2R18P* has a high level of identity with the functional *TAS2R42* gene and with the *TAS2R67P* pseudogene (66.59 and 67.02%, respectively) (Additional file 8: Figure S4). Pseudogenes derived from a very recent pseudogenization event (i.e. *TAS2R64P*) showed a much higher sequence similarity (91.32%) with their functional forms (i.e. *TAS2R48*), indicating ancient pseudogenization events at the *TAS2R6P* and *TAS2R18P* loci.

### Test of neutrality and mutation age estimates

**Figure 5:**
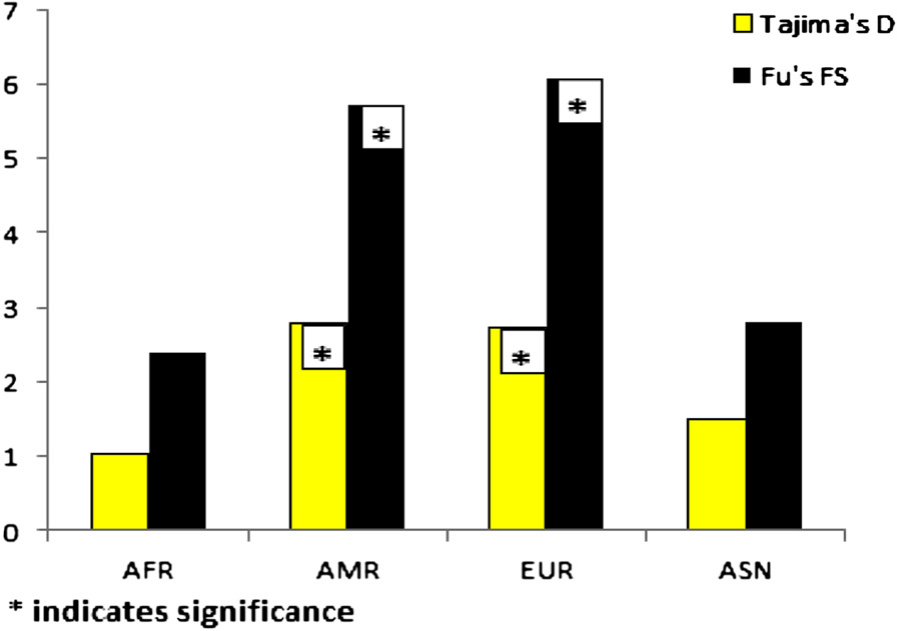
**Tajima’s D and Fu’s FS statistics for** ***TAS2R18P*** **in African, Asian, European and Latin American populations.**

Neutrality tests based on the analysis of frequency spectrum of variants were performed for each population. European and Latin American groups showed significantly positive values for both *TAS2R18P* Tajima’s D and Fu’s FS. Africans and Asians also showed positive values (1.04, 2.4 and 1.5, 2.8 respectively) for these statistics, although these did not reach significance (Figure 5). In contrast, *TAS2R6P* did not show evidence of deviation from neutral expectations in all the examined populations (p > 0.05). However, *rs62477710*, *rs10952507* and *rs6962558* polymorphisms in the adjacent *TAS2R5* gene showed significantly positive values of Tajima’s D and Fu’s FS (Additional file 9: Figure S5). The maximum frequency of derived mutations (MFDM) test, which uses tree topology to infer selection, showed similar results. In fact, both *TAS2R18P* and *TAS2R5* showed significant p-values (p = 9.16E-4 and p = 0.025, respectively), indicating that recent selection has acted on these loci. However, the p-values for this test were not significant for *TAS2R6P* (p > 0.05).

Finally, the ages of these variants were estimated using a coalescent-based method implemented in the GENETREE package. Estimated ages turned out to be considerably ancient. For SNPs in *TAS2R18P*, *rs2290318* was estimated to be 1,553,750 +/− 632,500 years old, while rs2290319 was estimated to be 1,113,750 +/− 495,000 years old, with a TMRCA of 3,520,000 +/−1,198,750 years. Similar values were also obtained for the *TAS2R6P* SNPs *rs11761380* and *rs1859645*, with age estimates of 1,382,462 +/− 326,856 and 1,600,637 +/− 421,558 years, and a TMRCA of 3,457,712 +/− 1,326,856 years.

## Discussion

A small number of studies have examined genes encoding functional bitter taste receptors and their corresponding pseudogenes from an evolutionary perspective [5,7,23,25,26]. In the present study, we focused on the recent evolutionary history of bitter taste receptor pseudogenes in modern humans. We found a modest level of variation and few haplotype blocks, as expected for pseudogenes in general. However, African populations showed a significantly reduced frequency of derived forms at *TAS2R6P* and *TAS2R18P* polymorphisms, at both the allelic and haplotypic level. Genomes of the archaic Neandertal and Denisova species also differed in the distribution of these derived alleles compared to modern human populations. In contrast to the usual pattern of human variation, the highest genetic diversity was found in non-African populations. Similarly, heterozygosity was higher in non-African populations. In addition, results from both population structure analyses and AMOVA demonstrated that most of variation was due to differences within populations. In fact, Europeans and Latin Americans fell into the same cluster and did not differ in allelic distribution, suggesting a similar pattern of SNP distribution in these two populations and highlighting the admixed nature of the 1000 Genomes American populations.

Previous work [5,7,23] indicated that the pseudogenization event of *TAS2R6P* is very old, predating the divergence of Antrhopoidea from Prosimians. The *TAS2R18P* pseudogenization event occurred later in the primate genealogy, but still prior to the appearance of the Hominidae family.

Our results were consistent with these findings: *TAS2R6P* variation patterns enabled the identification of a unique cluster grouping Prosimians, Hylobatidae and Hominidae families and high level of sequence similarity among primates (>95%), as well as between primates and Prosimians (>75%). *TAS2R18P* sequence similarity was lower between the mouse lemur and other primates (<70%) and the reconstructed phylogenetic trees showed longer branch lengths after the divergence of the two lineages. In addition, sequence similarity confirmed this view, showing a lower percentage (<70%) of identity between these two pseudogenes and their functional forms, when compared to the similarities between very recent pseudogenes and their functional forms (>90%).

The analysis of the genomic regions surrounding these pseudogenes showed that *TAS2R6P* polymorphisms *rs11761380* and *rs1859645* were in strong linkage disequilibrium (r^2^ > 0.9) with *rs62477710*, *rs6962558* and *rs6962558* SNPs of the functional *TAS2R5* gene, suggesting that evolutionary forces acting on this gene could have driven the allele differentiation at the TAS2R6P locus. To test this assumption, Tajima’s D and Fu’s FS tests were performed: these analyses showed that *TAS2R6P* did not show evidence of departure from neutral expectations while the neighboring *TAS2R5* gene, which shared more than 60% sequence similarity with *TAS2R6P*, showed significant positive values for both these statistics. Since positive values of these tests may indicate balancing selection or may be the result of the confounding effect due to demographic history [43,44], the maximum frequency of derived mutations (MFDM) test was applied to further investigate the evolutionary history of these regions. The results of this test indicate that *TAS2R6P* has not undergone recent selective pressures, whereas *TAS2R5* showed significant signatures of selection. This is consistent with balancing selection maintaining multiple alleles for long evolutionary times, and extending to maintain allele frequencies at closely linked neutral sites [45,46]. The application of this test to *TAS2R18* also showed significant signatures of selection and both Tajima’s D and Fu’s FS values were significantly positive at this locus in European and Latin American populations. This suggests that balancing selection potentially acted on the genes in these groups. In addition, these populations showed increased values of nucleotide diversity and heterozygosity in the *TAS2R18P* pseudogene compared to neighboring loci. Moreover, global F_ST_ values were unusually high compared to the typical range of 0.10 to 0.16 for estimated F_ST_ values in global populations [47,48], suggesting a high level of genetic differentiation among worldwide populations. These data are consistent with a scenario of balancing selection maintaining *TAS2R18P* alleles and enhancing genetic diversity at this locus in European and Latin American populations.

Variants at *TAS2R18P* showed no evidence of association with adjacent functional genes. This pseudogene carries a human-specific stop-codon that is shared among H. sapiens Neandertal and Denisova. In addition, the exonic region of this pseudogene carried four polymorphic insertions in the reading frame, upstream of the shared stop-codons and downstream of the human-specific one. These data suggest that these insertions may have shifted the human *TAS2R18P* reading frame, with a consequent acquired function of this gene, shortly after the homo-chimp divergence. Such an event, followed by balancing selection operating outside Africa, would produce the observed *TAS2R18P rs2290318* and *rs2290318* different allele distributions in human populations. The estimated ages of these mutations indicate that they arose after the divergence of humans from chimpanzee that occurred 7–8 million years ago [49]. We hypothesize that the human-specific stop codon located upstream of all these insertions represents a second inactivation, which happened before the split between H. sapiens, Neandertal and Denisova (from 400,000 to 800,000 years ago).

## Conclusions

Our results provide evidence for a dynamic role for *TAS2R18P* in primate evolution, suggesting that this locus may have acquired its function during the evolution of the human lineage, shortly after the homo-chimp divergence. This was followed by a much more recent deactivation due to the stop-gain mutation which was shared among modern humans, Neandertals and Denisovans. We speculate that this event may have been due to the disappearance of some bitter compound only found in Africa, which was specifically recognized by the product of this pseudogene, and that therefore the functionality of *TAS2R18P* was useful only in a given stage of human evolution.

## Availability of supporting data

The article does not report new empirical data since the analyzed sequences were already deposited at public databases, including the 1000 Genomes Project, the UCSC Genome Browser and the Ensemble Genome Browser (see the Methods section for further details).

## Additional files

Additional file 1: Table S1. Identified SNPs in the examined pseudogenes. Additional file 2: Figure S1. A) Haplotype blocks found on chromosome 7. B) Haplotype blocks found on chromosome 12. Additional file 3: Figure S2. Median-joining network of inferred haplotypes on A) *TAS2R6P* (H5) and B) *TAS2R18P* (H6) genes. Additional file 4: Table S2. Number of Polymorphic Sites (PS), Nucleotide Diversity (π) and Estimated Heterozigosity (EH) in the examined populations in A) *TAS2R6P* and B) *TAS2R18P.* YRI, Yoruba from Ibadan (Nigeria); LWK, Luhya from Webuye (Kenya); ASW, people with African ancestry from Southwest United States; IBS, Iberian populations from Spain; TSI, Tuscans from Italy; CEU, Utah residents with Northern and Western European ancestry; GBR, British from England and Scotland; FIN, Finnish; PUR, Puerto Ricans; CLM, Colombians from Medellin; MXL, people with Mexican ancestry from Los Angeles; JPT, Japanese from Tokyo; CHB, Han Chinese from Beijing; CHS, Han Chinese from Southern China. Additional file 5: Figure S3. Two dimensional scaling of FST distance matrix calculated for *TAS2R6P* in the examined populations. Additional file 6: Table S3. Sequence similarity among the studied species in A) *TAS2R6P* and B) *TAS2R18P* genes. Additional file 7: Table S4. LD values in *TAS2R6P* surrounding regions. Additional file 8: Figure S4. Cladograms based on comparisons between *TAS2R6P* and *TAS2R18P* and neighboring genes. Additional file 9: Figure S5. Tajima’s D and Fu’s FS statistic in African, Asian, European and Latin American populations for *TAS2R5 rs62477710,* *rs10952507* and *rs6962558* polymorphisms.
