# Supplementary material for: Genetic variation in taste receptor pseudogenes provides evidence for a dynamic role in human evolution
Source: BMC Evol Biol. 2014 Sep 13;14:198. doi: 10.1186/s12862-014-0198-8 (PMC4172856; doi:10.1186/s12862-014-0198-8)
Supplement: Additional file 1: Table S1. — Identified SNPs in the examined pseudogenes. [file 12862_2014_198_MOESM1_ESM.pdf]

**Table S1.** Identified SNPs in the examined pseudogenes.
CHR, chromosome; A, allele; MAF, minor allele frequency.

| CHR | SNP | A1 | A2 | MAF |
|---|---|---|---|---|
| 7 | *rs1859645* | G | A | 0.49 |
| 7 | *rs11761380* | C | A | 0.49 |
| 7 | *rs847923* | C | G | 0.41 |
| 7 | *rs6963925* | T | C | 0.28 |
| 7 | *rs6944279* | T | A | 0.27 |
| 7 | *rs10239143* | G | C | 0.22 |
| 7 | *rs4726624* | A | G | 0.22 |
| 7 | *rs12535592* | G | A | 0.17 |
| 7 | *rs73058722* | G | A | 0.17 |
| 7 | *rs34039200* | A | G | 0.12 |
| 7 | *rs10242038* | A | T | 0.03 |
| 7 | *rs117638090* | A | G | 0.01 |
| 7 | *rs78023269* | T | C | 0.01 |
| 12 | *rs34648613* | T | A | 0.43 |
| 12 | *rs7310047* | A | G | 0.40 |
| 12 | *rs2290318* | C | G | 0.40 |
| 12 | *rs2290319* | A | C | 0.40 |
| 12 | *rs2599394* | A | G | 0.39 |
| 12 | *rs2708319* | G | A | 0.39 |
| 12 | *rs2597985* | C | T | 0.39 |
| 12 | *rs11507728* | T | C | 0.29 |
| 12 | *rs68071847* | G | A | 0.24 |
| 12 | *rs11054092* | C | T | 0.24 |
| 12 | *rs11054093* | C | G | 0.24 |
| 12 | *rs11054094* | A | G | 0.24 |
| 12 | *rs4763599* | G | A | 0.24 |
| 12 | *rs17810798* | C | G | 0.24 |
| 12 | *rs11054095* | C | G | 0.24 |
| 12 | *rs11054097* | C | G | 0.24 |
| 12 | *rs11054096* | C | T | 0.23 |
| 12 | *rs61928603* | C | T | 0.22 |
| 12 | *rs35062230* | A | G | 0.21 |
| 12 | *rs319269* | C | A | 0.21 |
| 12 | *rs7296270* | A | T | 0.21 |
| 12 | *rs61928604* | C | T | 0.20 |
| 12 | *rs7975933* | C | T | 0.04 |
| 12 | *rs74062459* | G | A | 0.04 |
| 12 | *rs184006834* | A | G | 0.02 |
| 12 | *rs139903046* | T | A | 0.02 |
| 12 | *rs2597986* | C | T | 0.02 |
| 12 | *rs114830595* | A | G | 0.02 |
| 12 | *rs145446085* | C | T | 0.02 |
| 12 | *rs143552376* | T | G | 0.02 |
| 12 | *rs150917538* | G | A | 0.01 |
| 12 | *rs79017255* | C | T | 0.01 |
| 12 | *rs142359564* | T | C | 0.01 |
| 12 | *rs73260727* | G | T | 0.01 |
