# Supplementary figures and images for: Genetic variation in taste receptor pseudogenes provides evidence for a dynamic role in human evolution
Source: BMC Evol Biol. 2014 Sep 13;14:198. doi: 10.1186/s12862-014-0198-8 (PMC4172856; doi:10.1186/s12862-014-0198-8)

**Figure S1.** A Haplotype blocks found on chromosome 7.  
B Haplotype blocks found on chromosome 12.

**A**

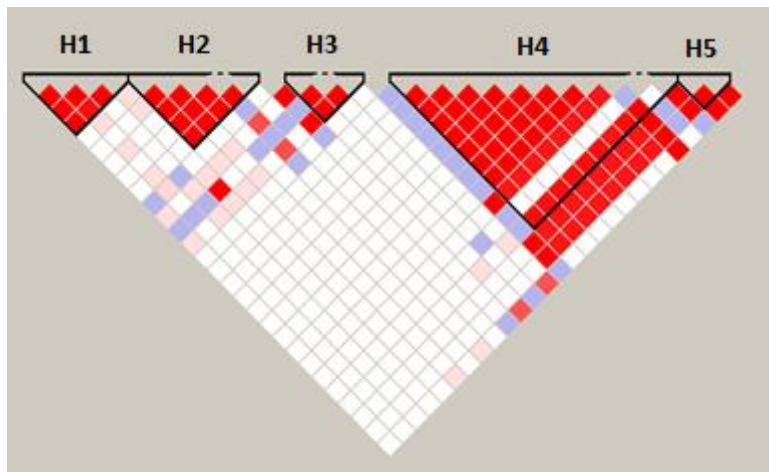

**B**

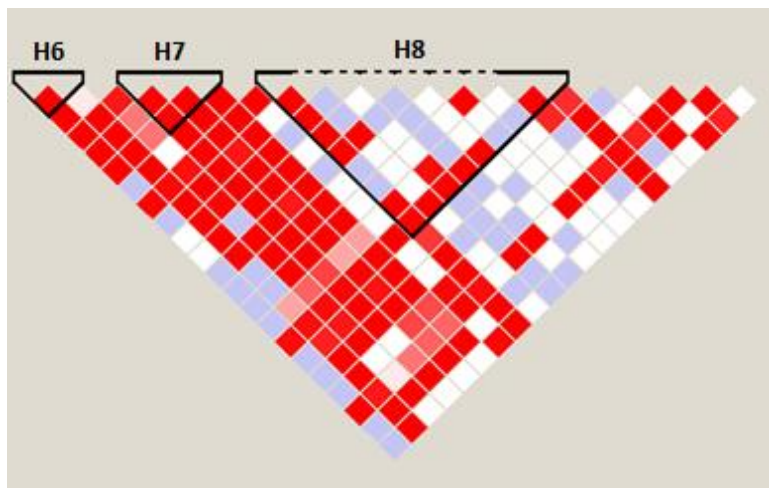

Supplement: Additional file 2: Figure S1. — A) Haplotype blocks found on chromosome 7. B) Haplotype blocks found on chromosome 12. [file 12862_2014_198_MOESM2_ESM.pdf]

**Figure S2.** Median-joining network of inferred haplotypes on A) *TAS2R6P* (H5) and B) *TAS2R18P* (H6).

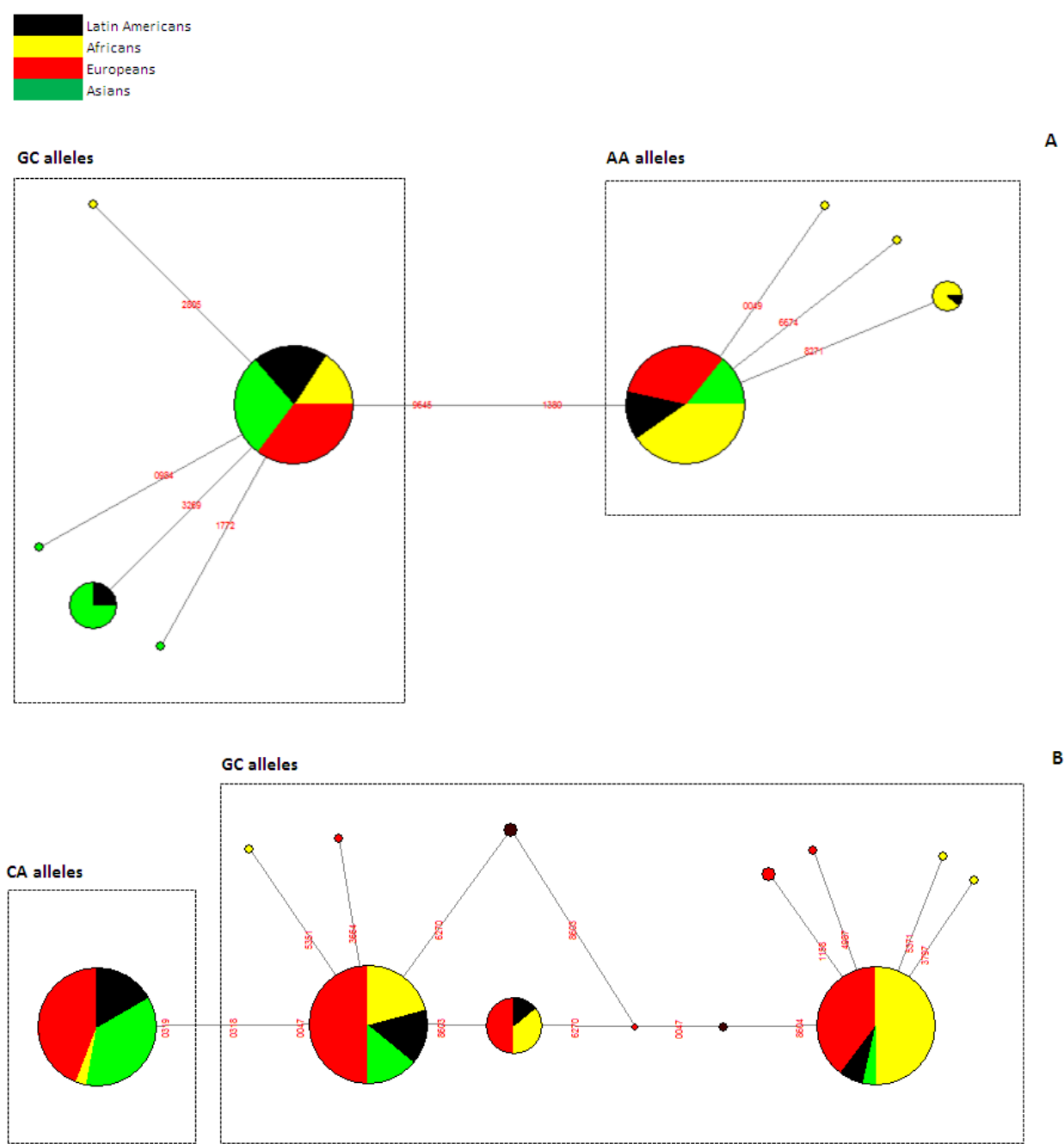

Supplement: Additional file 3: Figure S2. — Median-joining network of inferred haplotypes on A) TAS2R6P (H5) and B) TAS2R18P (H6) genes. [file 12862_2014_198_MOESM3_ESM.pdf]

**Figure S4.** Cladograms based on comparisons between *TAS2R6P* and *TAS2R18P* and neighboring genes.

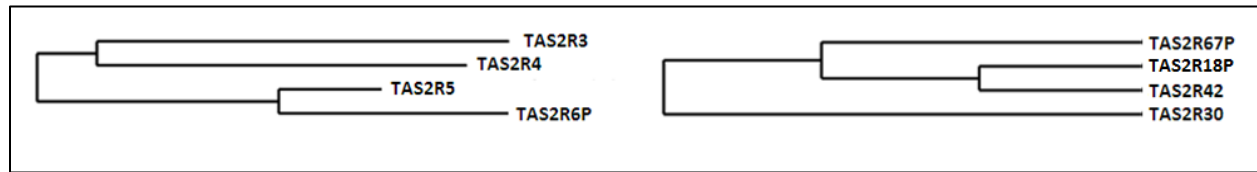

Supplement: Additional file 8: Figure S4. — Cladograms based on comparisons between TAS2R6P and TAS2R18P and neighboring genes. [file 12862_2014_198_MOESM8_ESM.pdf]
