# Supplementary material for: Genetic variation in taste receptor pseudogenes provides evidence for a dynamic role in human evolution
Source: BMC Evol Biol. 2014 Sep 13;14:198. doi: 10.1186/s12862-014-0198-8 (PMC4172856; doi:10.1186/s12862-014-0198-8)
Supplement: Additional file 4: Table S2. — Number of Polymorphic Sites (PS), Nucleotide Diversity (π) and Estimated Heterozigosity (EH) in the examined populations in A) TAS2R6P and B) TAS2R18P. YRI, Yoruba from Ibadan (Nigeria); LWK, Luhya from Webuye (Kenya); ASW, people with African ancestry from Southwest United States; IBS, Iberian populations from Spain; TSI, Tuscans from Italy; CEU, Utah residents with Northern and Western European ancestry; GBR, British from England and Scotland; FIN, Finnish; PUR, Puerto Ricans; CLM, Colombians from Medellin; MXL, people with Mexican ancestry from Los Angeles; JPT, Japanese from Tokyo; CHB, Han Chinese from Beijing; CHS, Han Chinese from Southern China. [file 12862_2014_198_MOESM4_ESM.pdf]

Table S2. Number of Polymorphic Sites (PS), Nucleotide Diversity (π) and Estimated Heterozigosity (EH) in the examined populations in A) *TAS2R6P* and B) *TAS2R18P*. YRI, Yoruba from Ibadan (Nigeria); LWK, Luhya from Webuye (Kenya); ASW, people with African ancestry from Southwest United States; IBS, Iberian populations from Spain; TSI, Tuscans from Italy; CEU, Utah residents with Northern and Western European ancestry; GBR, British from England and Scotland; FIN, Finnish; PUR, Puerto Ricans; CLM, Colombians from Medellin; MXL, people with Mexican ancestry from Los Angeles; JPT, Japanese from Tokyo; CHB, Han Chinese from Beijing; CHS, Han Chinese from Southern China

A

Population	PS	π	EH
ASW	4	0.51 +/- 0.03	0.46
LWK	4	0.36 +/- 0.03	0.35
YRI	5	0.37 +/- 0.04	0.32
CHB	4	0.46 +/- 0.03	0.41
CHS	4	0.47 +/- 0.03	0.43
JPT	3	0.46 +/- 0.03	0.42
CEU	3	0.53 +/- 0.01	0.50
FIN	3	0.51 +/- 0.01	0.50
GBR	3	0.49 +/- 0.01	0.49
IBS	2	0.49 +/- 0.05	0.49
TSI	3	0.53 +/- 0.01	0.50
CLM	3	0.53 +/- 0.01	0.50
MXL	4	0.56 +/- 0.02	0.49
PUR	5	0.57 +/- 0.02	0.50

B

Population	PS	π	EH
ASW	6	0.20 +/- 0.12	0.19
LWK	8	0.15 +/- 0.10	0.05
YRI	7	0.17 +/- 0.11	0.09
CHB	6	0.39 +/- 0.03	0.37
CHS	6	0.44 +/- 0.03	0.41
JPT	6	0.36 +/- 0.04	0.35
CEU	6	0.58 +/- 0.02	0.43
FIN	7	0.54 +/- 0.02	0.46
GBR	7	0.61 +/- 0.02	0.44
IBS	8	0.71 +/- 0.06	0.42
TSI	6	0.66 +/- 0.02	0.49
CLM	6	0.60 +/- 0.02	0.49
MXL	6	0.61 +/- 0.02	0.50
PUR	6	0.68 +/- 0.01	0.47
