# Supplementary material for: Genetic variation in taste receptor pseudogenes provides evidence for a dynamic role in human evolution
Source: BMC Evol Biol. 2014 Sep 13;14:198. doi: 10.1186/s12862-014-0198-8 (PMC4172856; doi:10.1186/s12862-014-0198-8)

Figure S3. Two dimensional scaling of F_{ST} distance matrix calculated for *TAS2R6P* in the examined populations.

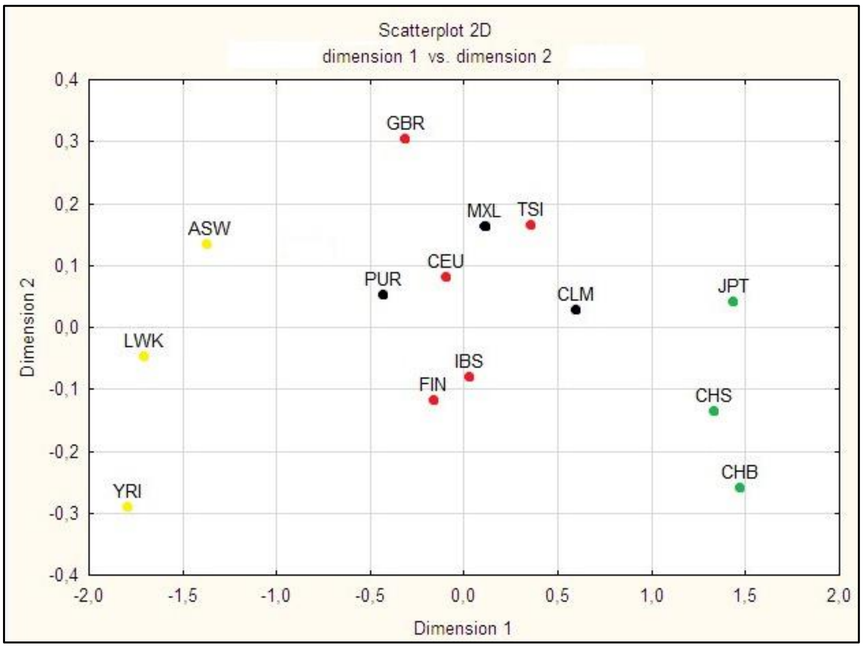

Supplement: Additional file 5: Figure S3. — Two dimensional scaling of FST distance matrix calculated for TAS2R6P in the examined populations. [file 12862_2014_198_MOESM5_ESM.pdf]
