# Supplementary material for: Genetic variation in taste receptor pseudogenes provides evidence for a dynamic role in human evolution
Source: BMC Evol Biol. 2014 Sep 13;14:198. doi: 10.1186/s12862-014-0198-8 (PMC4172856; doi:10.1186/s12862-014-0198-8)
Supplement: Additional file 6: Table S3. — Sequence similarity among the studied species in A) TAS2R6P and B) TAS2R18P genes. [file 12862_2014_198_MOESM6_ESM.pdf]

Table S3. Sequence similarity among the studied species in A) *TAS2R6P* and B) *TAS2R18P*.

A

Species1	Species2	%Similarity
sapTAS2R6P	sapTAS2R6P	99.99
sapTAS2R6P	sapTAS2R6P	99.99
chiTAS2R6P	gorTAS2R6P	98.91
sapTAS2R6P	gorTAS2R6P	98.79
sapTAS2R6P	chiTAS2R6P	98.67
chiTAS2R6P	gibTAS2R6P	97.09
gorTAS2R6P	gibTAS2R6P	96.97
sapTAS2R6P	gibTAS2R6P	96.73
gorTAS2R6P	oraTAS2R6P	96.62
chiTAS2R6P	oraTAS2R6P	96.49
sapTAS2R6P	oraTAS2R6P	96.37
gibTAS2R6P	oraTAS2R6P	96.01
gibTAS2R6P	lemTAS2R6P	77.48
chiTAS2R6P	lemTAS2R6P	77.24
gorTAS2R6P	lemTAS2R6P	77.17
sapTAS2R6P	lemTAS2R6P	77.01
oraTAS2R6P	lemTAS2R6P	76.61

B

Species1	Species2	%Similarity
sapTAS2R18P	denTAS2R18P	99.99
sapTAS2R18P	neaTAS2R18P	99.99
sapTAS2R18P	oraTAS2R18P	96.58
sapTAS2R18P	gorTAS2R18P	99.12
chiTAS2R18P	gorTAS2R18P	98.68
sapTAS2R18P	chiTAS2R18P	98.02
oraTAS2R18P	gorTAS2R18P	97.24
oraTAS2R18P	gibTAS2R18P	97.24
gibTAS2R18P	gorTAS2R18P	97.03
sapTAS2R18P	gibTAS2R18P	96.37
chiTAS2R18P	oraTAS2R18P	96.25
chiTAS2R18P	gibTAS2R18P	96.04
gorTAS2R18P	lemTAS2R18P	66.59
oraTAS2R18P	lemTAS2R18P	66.56
gibTAS2R18P	lemTAS2R18P	66.48
sapTAS2R18P	lemTAS2R18P	65.93
chiTAS2R18P	lemTAS2R18P	65.61
