# Supplementary material for: Genetic variation in taste receptor pseudogenes provides evidence for a dynamic role in human evolution
Source: BMC Evol Biol. 2014 Sep 13;14:198. doi: 10.1186/s12862-014-0198-8 (PMC4172856; doi:10.1186/s12862-014-0198-8)
Supplement: Additional file 7: Table S4. — LD values in TAS2R6P surrounding regions. [file 12862_2014_198_MOESM7_ESM.pdf]

Table S4. LD values in *TAS2R6P* surrounding regions.

**Gene1**	**SNP1**	**Gene2**	**SNP2**	**R2**
TAS2R6	*rs11761380*	*TAS2R5*	*rs62477710*	1
TAS2R6	*rs11761380*	*TAS2R5*	*rs10952507*	0.91
TAS2R6	*rs1859645*	*TAS2R5*	*rs6962558*	0.99
TAS2R5	*rs62477710*	*TAS2R5*	*rs10952507*	0.92
