# Supplementary material for: Genetic variation in taste receptor pseudogenes provides evidence for a dynamic role in human evolution
Source: BMC Evol Biol. 2014 Sep 13;14:198. doi: 10.1186/s12862-014-0198-8 (PMC4172856; doi:10.1186/s12862-014-0198-8)

Figure S5. Tajima's D and Fu's FS statistic in African, Asian, European and Latin American populations for *TAS2R5 rs62477710*, *rs10952507* and *rs6962558* polymorphisms.

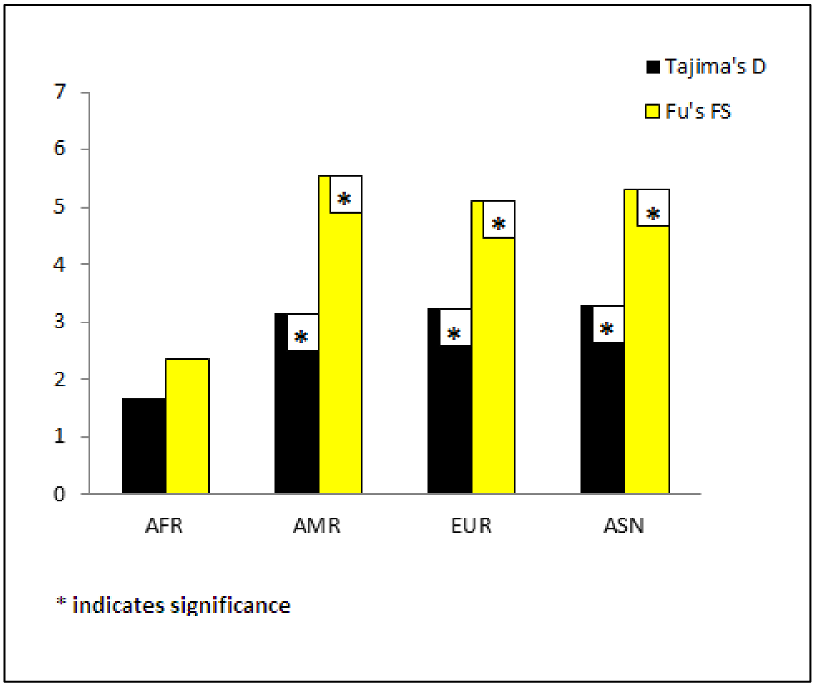

Supplement: Additional file 9: Figure S5. — Tajima’s D and Fu’s FS statistic in African, Asian, European and Latin American populations for TAS2R5 rs62477710, rs10952507 and rs6962558 polymorphisms. [file 12862_2014_198_MOESM9_ESM.pdf]
